# Feasibility and acceptability of a school-based Group Motivational Interviewing intervention to reduce sugar-sweetened beverages among young people in East London: DISS feasibility study

**DOI:** 10.1136/bmjph-2025-003961

**Published:** 2026-04-13

**Authors:** Huda Yusuf, Elizabeth Steed, Maria Josefina Valenzuela

**Affiliations:** 1 Centre for Dental Public Health and Primary Care, Institute of Dentistry, Queen Mary University of London, London, UK; 2 Wolfson Institute of Population Health, Centre for Primary Care, Queen Mary University of London, London, UK

**Keywords:** Primary Prevention, Public Health, Obesity

## Abstract

**Introduction:**

Young people consume sugar-sweetened beverages (SSBs) regularly which increases their risk of obesity and dental caries. School-based interventions can reduce the consumption of SSBs. This study aimed to assess the feasibility and acceptability of a Group Motivational Interviewing intervention delivered by teachers to reduce SSB consumption among 12–13 year-olds in secondary schools in East London to prevent weight gain.

**Methods:**

A feasibility study with an embedded exploratory mixed-methods design was conducted. Participants included children aged 12–13 years old and five teachers. Intervention was co-developed with teachers and young people, which included three components: Group Motivational Interviewing training to teachers to deliver two sessions to young people, a mobile application for young people was developed to support them with behaviour change and a resource pack for parents/carers. The main outcome measures at baseline and follow-up were anthropometric, dietary and lifestyle behaviours. Feasibility measures included recruitment and retention rates, intervention delivery and acceptability. Thematic analysis was used to analyse the qualitative data and descriptive analysis for the quantitative data.

**Results:**

One secondary school was recruited despite attempting to recruit six schools, and a second school was recruited to co-develop the intervention. Five teachers, 49 young people and their parents (n=26) were recruited in one school. At follow-up (n=47), SSB consumption decreased, and water consumption increased, but this was based on a limited dietary recall sample. The main challenge was the recruitment of schools due to limited school resources and organisational factors. In terms of acceptability, once young people were recruited, they reported positive attitudes towards the intervention whereas teachers found it less feasible due to structural constraints.

**Conclusions:**

Although the intervention was acceptable and feasible in school settings, there were significant challenges in recruitment of schools. The intervention was more acceptable for students than teachers. This study showed the importance of conducting feasibility studies prior to a large trial.

**WHAT IS ALREADY KNOWN ON THIS TOPIC**

- There is an increased prevalence of young people consuming sugar-sweetened beverages (SSBs), which is a risk factor for both obesity and dental caries.
- School-based interventions can be effective in reducing SSB intake, but there is limited evidence in ethnically diverse and deprived populations.

**WHAT THIS STUDY ADDS**

- A multicomponent intervention based on the WHO Health Promoting Schools Framework was co-developed with young people and school staff.
- The intervention was more feasible and acceptable to young people than teachers in an East London secondary school.
- There were significant challenges in recruiting schools primarily due to limited resources and organisational factors.

**HOW THIS STUDY MIGHT AFFECT RESEARCH, PRACTICE OR POLICY**

- Co-developing the intervention with young people and teachers ensured that it is relevant and acceptable.
- The study highlights the critical importance of conducting a feasibility study before a larger-scale trial to identify and address practical barriers, particularly related to school and pupil recruitment.

## Background

 The increased consumption of sugar-sweetened beverages (SSBs) has been identified as a key driver of obesity among children and adolescents, which is a global public health burden. Between 1990 and 2018, the intake of SSBs among individuals aged 3–19 years increased by 23%, globally.^1^ In England, approximately 70% of individuals aged 11–18 years regularly consume SSBs, representing the largest single source of dietary sugars within this age group.^2^ There are associated inequalities in obesity prevalence by deprivation and ethnicity, which are attributable to the wider social determinants of health. Children from disadvantaged backgrounds and those from Asian (44.3%), black (43.2%) and other ethnic backgrounds (43.8%) disproportionately bear the burden of higher rates of weight gain.^3^

Locally, Tower Hamlets is one of the most deprived and ethnically diverse populations in England with nearly half of children (48%) living in poverty (an income below 60% of the UK median).^4^ There is a high prevalence of obesity among 10-year-olds (43.1%) when compared with the national average (36.6%).

One of the local challenges is the obesogenic environment and readily available fast food and drinks outlets which can compromise young people’s dietary behaviours. A systems-wide approach is required to tackle childhood obesity in which health in all policies is embedded within all sectors.^5^ This includes school-based interventions supporting children and young people to maintain healthier weights. There has been limited research conducted among young people in secondary schools to reduce their SSB intake in England.^6^

Adolescence is a critical developmental period in which lifelong health behaviours are established including the intake of SSBs. Therefore, interventions during this period can have significant impacts on overall health and well-being in the long term.

Teachers have an important role in promoting health; however, they may not have the specific skills in behaviour change. Psychological techniques such as Group Motivational Interviewing (GMI) could support teachers in facilitating healthy behaviour change in the classroom.^7^ GMI has been used effectively in tackling adolescents’ drug and alcohol use, but not for dietary sugar reduction to reduce obesity. GMI is ideal for adolescents, because it collaborates with them, eliciting their own ideas about healthy behaviours, thereby respecting individual autonomy.

The aim of this study was to assess the feasibility and acceptability of an obesity intervention delivered by teachers to reduce SSB consumption and prevent weight gain among 12–13 year-olds in secondary schools using GMI. The study acronym DISS sugary drinks in schools was created by young people. It is important to conduct feasibility studies before proceeding to a full-scale trial to determine whether a study can be practically delivered in real-world settings and refining the design of the study before investing in substantial time and resources.

## Methods

### Study design and setting

The DISS intervention was aimed at reducing sugar intake among young people attending secondary schools in East London. It was evaluated using a feasibility study design with clinical and subjective measures as well as embedded process measures assessing feasibility and acceptability among young people and school staff. Age-appropriate information sheets were developed, and consent/assent was sought from teachers, young people and their parents. The classroom intervention was delivered to all young people, irrespective of having consented to the data collection process.

All data were processed and stored in accordance with the 2018 Data Protection Regulation (GDPR) principles and the Queen Mary University of London’s Information/Data Governance Policy.

### Sample size

This study was not adequately powered to detect changes in any of the primary and secondary outcomes. It assessed practicality and acceptability of the intervention among study participants. Four to six secondary state schools were targeted for recruitment with 180 pupils in each school.

### Study participants and recruitment

Schools were eligible if they were state-funded secondary schools located in East London. Within schools, participants included secondary school teachers, young people aged 12–13 years old (year 8) irrespective of their SSB intake, and their parents.

All state-funded secondary schools in East London were approached by email to take part in the study. The study was promoted through head teachers’ newsletters as well as attending headteacher’s forum to recruit schools.

The schools provided gateway consent for gaining access to young people and their parents and carers. Young people and their parents were invited through the school’s newsletter, posters displayed in the school and school staff who supported recruitment of students.

### Intervention

The development of the intervention has been reported in a protocol paper.^8^

The intervention consisted of three components based on the WHO Healthy Schools Framework.

#### Upskilling teachers in GMI and inclusion of the intervention into the PSHE education within the curriculum

The authors developed the content of the intervention in line with national and professional guidelines and existing literature. The intervention aimed at reducing SSB intake among young people by incorporating the intervention sessions into the curriculum. A clinical psychologist developed the GMI intervention to be delivered in a classroom setting. A manual was developed to standardise the intervention which integrates the public health guidance into the GMI intervention.^9^ The manual included the following domains: summary of the study, health profiles of young people living in the area and the impacts of SSB on general and oral health, GMI, delivery of the two sessions, activities and outcomes. The intervention was designed to be delivered in two sessions in a term integrated into science or personal, social, health and economic (PSHE) scheduled lessons.

The authors and the clinical psychologist delivered training to six physical activity teachers over two 3-hourly sessions. Teachers were trained in GMI and healthy eating and physical activity. The manual was provided to teachers to deliver the intervention over one term.

#### A mobile application for young people to support positive behaviour change (DISH DASH)

Young people’s advisory panel collaborated with the research team to co-develop the mobile application, so it is relevant and addresses the needs of young people. The panel supported the game development element of the application as well as the content of the health resources. A software company with expertise in developing mobile applications developed the Dish Dash app with feedback from young people. A user pathway was developed alongside evidence-based messages to promote reduction of SSB consumption and replacing it with water. Young people were given access to a mobile application based on Motivational Interviewing principles providing positive health messages before proceeding to play the game. This facilitated young people’s engagement with the study by having a leadership game board where participants competed with each other.

#### A health booklet for parents

Parents were provided with evidence-based health resources to translate the in-school intervention to the home setting, based on national guidelines to support their children to reduce SSB consumption and promote physical activity within the home environment.

### Patient and public involvement

The original research plan was informed by secondary school pupils, headteachers and a member of the PSHE Association outside the study area. The intervention was co-developed with young people and teachers. A teacher’s advisory panel and a young people’s advisory panel were set up in a different school to facilitate the collaborative development of the intervention. The panels provided advice on shaping the content and the structure of GMI sessions to ensure they were age-appropriate and aligned with school schedules. A final study event was hosted at the university to which local authorities, selected secondary school headteachers and pupils were invited and the schools who had participated in the study to disseminate the findings.

### Process evaluation

The process evaluation used for this feasibility study was informed by the MRC framework for conducting process evaluation.^10^ The domains assessed included recruitment and retention rates, dose delivered, barriers and facilitators to intervention implementation, and acceptability among teachers and young people.

#### Recruitment and retention

Both quantitative and qualitative data were collected to assess recruitment of participants, including assessing the feasibility of gaining consent from participants. Quantitative data were collected to determine recruitment, retention and attrition rates.

#### Dose of GMI delivered

The quantity of the intervention was assessed by the number and duration of sessions. The research team could not observe teachers in delivering the intervention.

#### Acceptability, barriers and facilitators

The acceptability, barriers and facilitators were explored through questionnaires and focus groups with young people and school staff.

#### Piloting primary and secondary health outcomes for the main trial

Anthropometric measures, dietary measures and young persons’ questionnaires were collected at baseline and at 6 months.

##### Dietary behaviours

Change in young person’s mean daily consumption of SSB, defined as the mean daily total volume (mL) and frequency (number of drinks per day), was collected through three repeated 24-hour dietary recalls at baseline and follow-up using Intake24.^11 12^

##### Anthropometric measures

Height without shoes was measured to the nearest 0.1 cm, using the portable Leicester height measure and weight in light clothing was measured to the nearest 0.1 kg on calibrated portable electronic scales (Seca 770). Waist circumference (WC) was measured at 2 cm above the navel. UK reference curves (above 85th centile: overweight and above the 95th centile: obese). z-scores were calculated for body mass index (BMI) and WC using the LMS method.^13^

##### Physical activity

A brief baseline and follow-up questionnaire was administered to young people to assess their physical activity.

### Data sources and data collection

#### Quantitative data

##### Young person’s baseline and follow-up questionnaires

A brief baseline and follow-up questionnaire were administered to young people which collected demographic data (age, sex, ethnicity and postcode), intake of SSBs, physical activity and self-efficacy questions to change SSB intake (onlinesupplemental files 1  2).

##### Parent’s baseline and follow-up questionnaires

Parents completed a questionnaire at baseline and follow-up that collected data about demographics (sex, employment status and education level), eating habits and physical activity (onlinesupplemental files 3  4).

### Qualitative data

#### Focus groups

Focus groups were conducted to explore participants’ perspectives on the study including the intervention and study implementation. This method was chosen to ensure interactive discussion ensuring a range of perspectives is considered. Two separate focus groups, one with teachers (n=4) and the other with young people (n=6), were conducted by the research team. The researchers recognised the power dynamics present during the focus groups and ensured that they had an open approach to the discussions. Recruitment of young people was based on a purposive sample according to gender and ethnicity. Topic guides were developed for each focus group to explore the acceptability, barriers and facilitators of each component of the intervention and the data collection process (online supplemental file 5). Positive and written consent was obtained from all participants.

#### Incentives

Incentives were offered to increase the recruitment rates and data collection involvement. Schools taking part in the study were offered a £150 voucher, young people a £5 voucher at baseline and £5 at follow-up, teachers a £30 voucher and parents a £25 voucher.

### Data management

Feasibility and acceptability of the intervention were assessed using an explanatory sequential mixed-methods approach.^14^

#### Quantitative data analysis

Data were imported into Stata V.18 MP for analysis. Descriptive analyses were used to describe baseline and follow-up characteristics of study participants and parents. Means and SD were calculated for continuous variables. There was no inferential statistics conducted.

#### Qualitative data analysis

Verbatim anonymised transcripts of the audio-recorded focus groups were analysed using a thematic analysis using an iterative and cyclical process.^15^ Initial codes were captured and reviewed alongside the topic guide to identify the broader themes. A thematic chart was developed. The data were re-examined, and the categorisations were refined in order to ensure that a logical and consistent pattern was being ensued.

## Results

### Recruitment and retention of secondary schools and young people

One school was successfully recruited to take part in the intervention, while a second school was recruited to inform the advisory panel to co-design the intervention. Of the 237 young people in 9 form classes who fulfilled the criteria, 50 provided consent/assent both from the young person and their parent/carer to take part in the measurements and data collection process (recruitment rate 21.09%). The baseline data collection was completed by 49 young people (one was absent on the 3 days of data collection at the school), and 26 parents.

**Figure 1:**
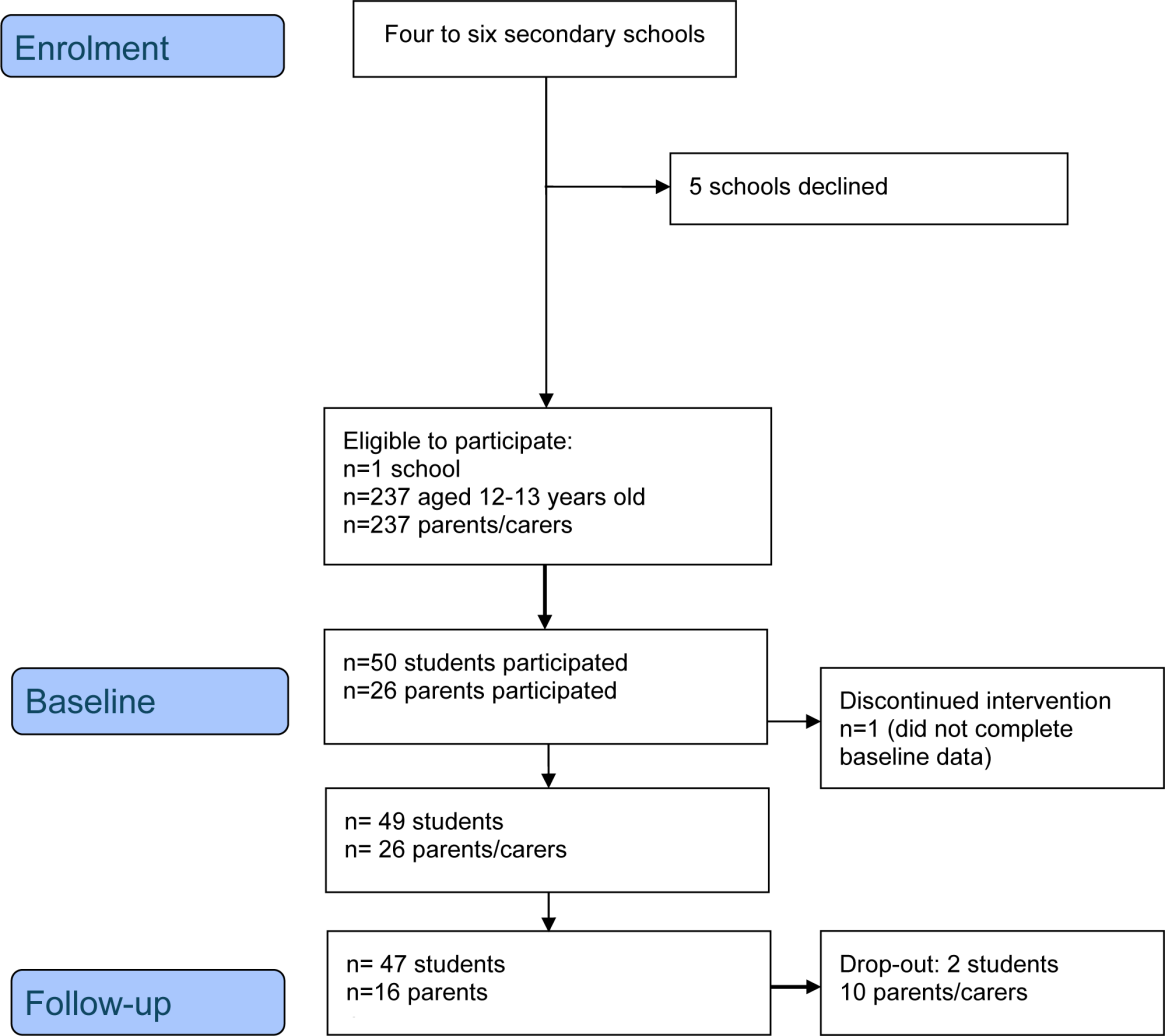
CONSORT flow chart for DISS study. CONSORT, Consolidated Standards of Reporting Trials.

Retention: The follow-up data collection was completed by 47 young people (one was no longer a student and two were absent on the day of data collection) and 16 parents, resulting in a retention rate of 92% and 61.53%, respectively (figure 1). For teachers, five completed the training and remained at follow-up (one was no longer a staff member at the school, a retention rate of 80%).

### Baseline characteristics of the sample

There were more boys than girls in the sample and 72% were aged 12 years (table 1). More than two-thirds of the sample of young people identified themselves as Asian/Asian British, followed by 14.29% as black/African/Caribbean, 10.20% as mixed ethnic backgrounds and a small minority 6.12% and 4.08% as white or other ethnic group, respectively. One third of parents did not hold a formal education qualification, and 23.08% of parents held a university degree. More than three-quarters of parents (84.62%) were in receipt of government benefits.

**Table 1:** Demographic characteristics of young people and their parents

Demographic variables	Frequency	Percentage (%)
*Young people, N=49*		
Sex		
Female	14	28
Male	35	72
Age at baseline		
12 years	36	72
13 years	13	28
Ethnicity		
White	3	6.12
Mixed/multiple ethnic groups	5	10.20
Asian/Asian British	32	65.31
Black/African/Caribbean/black British	7	14.29
Other ethnic group	2	4.08
*Parents, N=26*		
Sex		
Female	12	46.15
Male	14	53.85
Age at baseline		
<30 years*	5	26
30–39 years	3	11.53
40–49 years	10	38.46
>50 years	8	30.76
Highest levelof education		
No qualifications	8	30.77
GCSE or O Level	4	15.38
A Level or AS Level	1	3.85
BTEC/NVQ Level 1 or 2	3	11.54
BTEC/NVQ Level 3	1	3.85
Higher national diploma	2	7.69
University degree	6	23.08
Other	1	3.85
Benefits		
In receipt of benefits	22	84.62
No benefits	4	15.38

* Some parents put their child’s DOB or the date of the survey.

AS, Advanced Subsidiary Level; GCSE, General Certificate of Secondary Education; NVQ, National Vocational Qualification.

At baseline, teachers (3 men and 2 women) described their job positions as physical education (PE) teachers (n=4) and one director of sports.

### Young people’s health outcomes at baseline and follow-up

#### Dietary intake

Only 10 students completed the Intake24 survey at baseline and 10 at follow-up. Results demonstrated that the mean intake of sugary drinks—including fizzy drinks and fruit juices with natural or added sugars at baseline was 279.36 mL/day (SD=168.69), which decreased to 176.73 mL/day (SD=32.09) at follow-up. However, this was based on a limited dietary recall sample and therefore results need to be interpreted with caution. On the other hand, water intake increased, from 261.30 mL/day (SD=41.81) at baseline to 278.57 mL/day (SD=185.10) at follow-up (table 2). However, milk consumption remained relatively unchanged.

**Table 2:** Comparison of mean daily SSB consumption at baseline and follow-up among young people (data from Intake24)

Drink type	Baseline (n=10), mean (SD)	Follow-up (n=10), mean (SD)
*Mean daily consumption of sugary drinks (mL/day)*		
Fizzy drinks, fruit juices (with natural or added sugars)	279.36 (168.69)	176.73 (32.09)
Milk (mL/day)	247.07 (108.57)	242.76 (169.96)
Water (mL/day)	261.30 (41.81)	278.57 (185.10)

SSB, sugar-sweetened beverage.

Sugary food intake was explored using a survey questionnaire. At baseline, 65.3% of participants reported consuming chocolates and sweets two or more times per week, with 32.65% consuming them 2–4 days per week and a further 32.65% consuming them more than 5 days per week. At follow-up, this proportion slightly decreased, with 60.87% reporting consumption two or more times per week and 23.91% reporting intake more than 5 days/week (table 3). Fruit intake remained relatively stable from baseline to follow-up.

**Table 3:** Comparison of dietary habits at baseline and follow-up among young people (self-reported data from questionnaire)

How often do you eat or drink	N	Never or less than once a week	Once a week	2–4 days a week	More than 5 days a week
Chocolates and candy					
Baseline	49	9 (18.37%)	8 (16.33%)	16 (32.65%)	16 (32.65%)
Follow-up	46	8 (17.39%)	10 (21.74%)	17 (36.96%)	11 (23.91%)
Fizzy drinks, fruit juice or soft drinks like squash					
Baseline	48	14 (29.16%)	13 (27.08%)	13 (27.08%)	8 (16.66%)
Follow-up	47	14 (29.79%)	8 (17.02%)	17 (36.17%)	8 (17.02%)
Vegetables					
Baseline	49	5 (10.20%)	8 (16.33%)	8 (16.33%)	28 (57.14%)
Follow-up	46	2 (4.34%)	15 (32.61%)	6 (13.04%)	23 (50%)
Fruits					
Baseline	49	4 (8.16%)	2 (4.08%)	16 (32.65%)	27 (55.10%)
Follow-up	47	1 (2.13%)	3 (6.38%)	19 (40.43%)	24 (51.06%)

#### Anthropometric measurements

There was little change in BMI z-scores from 0.74 (SD 1.20) to 0.75 (SD 1.31) and WC z-scores from 1.04 (SD 1.18) to 1.17 (SD 1.09) scores, from baseline to follow-up, respectively (table 4). This needs to be interpreted with caution as we would not expect significant changes over a 6-month period, and the sample size was small.

**Table 4:** Comparison of anthropometric measures and physical activity at baseline and follow-up among young people (self-reported data from questionnaire)

Characteristic	Baseline (n=49), mean (SD)	Follow-up (n=47), mean (SD)
Anthropometric measures		
BMI	20.79 (3.97)	21.51 (4.32)
BMI z scores (mean, SD)	0.74 (1.20)	0.75 (1.31)
WC	71.87 (10.85)	73.97 (10.3)
WC z scores (mean, SD)	1.04 (1.18)	1.17 (1.09)
Physical activity Moderate to vigorous physical activity		
Days of activity over past 7 days	3.53 (1.77)	4.41 (1.95)
Hours a week of exercise in free time	3.51 (3.08)	3.49 (2.40)
Sedentary behaviour		
Hours per day of watching TV		
(a) Weekdays	2.75 (3.05)	2.00 (1.86)
(b) Weekends	3.42 (2.68)	3.52 (2.74)
Hours per day playing on a game console, social network sites or computer in general		
(a) Weekdays	2.03 (1.64)	2.60 (1.86)
(b) Weekends	3.23 (2.57)	4.09 (2.13)

BMI, body mass index; WC, waist circumference.

#### Physical activity

In terms of physical activity, there was a slight decrease from baseline to follow-up in number of days (3.53 (SD 1.77) to 4.41 (SD 1.95)). Sedentary behaviour decreased during weekdays from baseline to follow-up and was maintained during the weekends (table 4). However, use of gaming consoles increased during the same time.

### Process evaluation

#### Recruitment and retention of schools and young people

Recruitment of schools was challenging as the research study was implemented shortly after the COVID-19 pandemic. Out of the 4–6 schools initially targeted for recruitment, only one school accepted the invitation to participate in research. Feedback from teachers indicated that resources and capacity, teacher motivation and autonomy in timetabling and teaching influenced recruitment of schools.

Young people and teachers reported that parental influences, motivation and incentives were key factors in recruiting them as study participants.

Whether their parents didn’t want to be involved, whether they didn’t want their kids getting measured, whatever it is, which we do find obviously now with that being in charge of a year group rather than just teaching PE, a lot of parents are quite reluctant to receive any sort of guidance (T3)

#### Dose of GMI intervention delivered

In total, 18 GMI sessions were delivered by five PE teachers. Each young person received two sessions during school time. The first session lasted around 40 min and the second 15 min.

#### Acceptability of the GMI intervention

The GMI sessions were well accepted by young people. Young people reported that the number of GMI sessions was exactly right or would have preferred more sessions. Likewise, the length of the sessions was positively perceived, with 85% reporting that the length was right or would have been happy with longer sessions. Furthermore, 90.48% said that they would recommend the sessions to a friend. Overall, 42.5% of young people felt comfortable talking openly with their teacher during the GMI sessions at their school, they felt that the teacher listened to them, and the sessions were useful (table 5).

**Table 5:** Young people’s views on the GMI sessions

Views on the GMI sessions	N	Disagree	Neutral	Agree
I felt I could talk openly with my PSHE teacher during the sessions (on sugary drinks)	40	5 (12%)	18 (45%)	17 (42.50%)
I felt that my teacher listened to me	41	2 (4.88%)	20 (48.78%)	19 (46.35%)
Overall, I felt the sessions (on sugary drinks) were useful to me	41	2 (4.88%)	21 (51.22%)	18 (43.91%)
I felt comfortable that the sessions (on sugary drinks) took place in the school	41	2 (4.88%)	19 (46.34%)	20 (48.76%)

PSHE (personal, social, health and economic) education

GMI, group motivational interviewing.

In focus group discussions, students reported that schools were an acceptable setting to deliver the intervention, which was congruent to findings from focus groups with teachers.

It’s perfect (to do the intervention) in schools. (Pu 1)

I think it’s the right setting because I think if you do it outside of like, first of all, you’ve got a massive cohort of kids there that you can gain access to. (T2)

Young people felt that their health-related behaviours could be changed by highlighting the associated risks and providing the young person with SMART goals to support this.

What can I learn from you in terms of having that healthy diet and regular physical activity by showing the risks of what could happen? (Pu 2)

Although young people responded positively to the intervention and appreciated the autonomy embedded in GMI, the teachers had challenges in delivering the intervention. One of the key reasons is the inherently formal teacher-student relationships which do not foster pupil autonomy in practice. This was more pronounced among male students.

So as much as they’re right, I think in an environment, a school environment, it’s hard to switch from what they’re used to in a like teacher student relationship than what they would be used to if it was potentially an external person. (T2)

### Barriers and facilitators of the GMI Intervention

#### Achieving healthy behaviours

There was a consensus between perspectives of young people and teachers regarding the significant influence of the wider environment on healthy behaviours and the availability of unhealthy food and drinks can pose as a barrier for adopting such behaviours. At times, there was a sense of fatalism considering the environmental influences on behaviours.

So in terms of physical activity and healthy eating, because we know it’s really, really hard because we’re surrounded by junk food. (Pu 1)

You get students that we could have this afternoon doing PE lessons and then as soon as they’ve left the school, you can see them outside the school with like in the KFC shop and they’ve got like fried foods. (T1)

Cost was also an important factor in replacing SSBs with water. Both teachers and young people agreed that the cost of water was prohibitive when compared with the lower cost of SSBs.

At the end of the day, they’re not going to go and buy a bottle of water which is going to cost £1.20 when they can buy a Boost bottle which is 89p (T2)

The findings highlight that the wider environment has significant influences on children and young people’s behaviours.

## Discussion

This was an innovative feasibility study in using GMI in a school setting to reduce SSB intake among young people in a deprived and ethnically diverse area of London. The findings highlight challenges with recruitment of schools and young people, which was a significant barrier to intervention delivery. Although the intervention was acceptable, the influence of the wider environment in supporting healthy behaviour change should not be under-estimated.

Recruitment of schools was challenging despite repeated efforts through a variety of channels including local authorities, education and public health departments, PSHE leads, school to school advocacy and direct contact with schools. The COVID-19 pandemic had significant impacts on schools, staff and pupils, affecting both physical and mental health as well as educational attainment within the context of limited resources. This aligns with findings from the current literature.^16 17^ Furthermore, there is evidence that there are challenges in the implementation of school-based interventions which include community level factors, provider factors including staffing, resources, time, staff motivation and leadership, and individual level factors.^18^ This is reflected in the findings for the current study in terms of difficulties in recruitment of schools due to school staff being overwhelmed with teaching commitments and delivery of the curriculum. This means that schools and staff have limited autonomy to be involved in wider health promotion activities. Recruitment of schools was limited and this was despite the research team consulting with local schools and young people to inform the study design when setting up the study.^17^

As schools act as gatekeepers, the research team relied on them to allow the research team to interact with young people and their parents. The recruitment rate for young people (21.09%) and their parents also proved to be difficult. This is multifactorial and could be influenced by demographic factors such as socioeconomic status and ethnicity, motivation, school-related factors and research study implementation factors including communication and relevance of the topic.^19 20^ A large proportion of parents were from Asian/Asian British backgrounds and in receipt of benefits which may indicate a population with competing priorities. Children from more deprived backgrounds are at a higher risk of overweight and obesity.^4^ This aligns with findings from other studies. Although this study is not generalisable to the UK population as a whole, it provides valuable insights into conducting research in underrepresented groups and their associated burden of overweight and obesity.

Although the intervention did not assess effectiveness, it resulted in a decrease in mean daily sugary drinks intake and a slight increase in water intake, suggesting a positive change in the right direction. These findings align with previous research demonstrating that school-based interventions can influence SSB intake.^6^ However, the self-reported data on sugary food intake also showed a slight decrease, while physical activity levels increased and sedentary time on weekdays decreased. As there was a relatively short follow-up period, it was not expected to see changes in anthropometric measurements.^21^

### Acceptability, barriers and facilitators to intervention implementation

Young people were satisfied with the intervention, finding the GMI sessions helpful and valuing the sense of autonomy that it provided. However, teachers had a different view in which GMI was perceived to be difficult to achieve in a classroom as they attempted to maintain discipline. This may indicate changes to pedagogical approaches to teaching methods from traditional didactic approaches to one which promotes collaborative approaches. Getting this right balance between maintaining discipline and support in inner city London schools while facilitating individual autonomy is challenging. In a previous study, pupils reported that teachers preferred non-collaborative approaches to teaching whereas young people preferred some autonomy.^22^ However, evidence has shown that sessions where students are facilitated to explore attitudes, beliefs and experiences can be conducive to promoting competency-based learning, potentially resulting in healthy behaviour change.^23^ A major barrier was the challenge teachers faced in transitioning from their traditional, formal roles to a more collaborative and autonomy-supportive approach, particularly with male students. This highlights a critical need for more intensive training and ongoing support for teachers to effectively implement GMI-style interventions in schools.

The high acceptability of the intervention among young people is a key strength of this study. The positive feedback on session length, number of sessions and the school setting itself provides strong evidence that this model is well-received by the target population. Students appreciated the autonomy embedded in the GMI approach, which is a crucial facilitator for engagement and behaviour change.^24^ Young people engaged with the mobile application, the game and accessed health information and found it to be acceptable. This aligns well with other studies which have demonstrated that mobile applications can be conducive to healthy behaviour change.^25^

Both young people and teachers highlighted the influence of the wider environment, with the pervasive availability of cheap, unhealthy food and sugary drinks acting as a major barrier to positive behaviour change. The lower cost of SSBs relative to water illustrates the influence of commercial determinants as a powerful driver to negative behaviours.^26^ Despite having a fiscal measure of SSB tax in England, the cost of purchasing SSBs remained lower than that of water.

### Study limitations

There were several limitations to this study. The unsuccessful recruitment of 6 secondary schools with high population health needs highlighted the challenges in conducting research among an ethnically diverse and deprived populations. Recruitment of schools was central to the feasibility of conducting this study, as schools acted as gatekeepers for young people. Despite attempting to recruit schools by a variety of methods, many schools seemed to be overwhelmed by the demands of returning to normal operations following the disruptions caused by COVID-19.

Once a school was recruited, there were challenges in engaging with children and young people and their parents. There were significant challenges in data collection, especially dietary recalls, and also given the small sample size, it was difficult to make any inferences in terms of behaviours and anthropometric measures.

Teachers were receptive to GMI but reported challenges with its application. They found the concept of the study to be facilitative of promoting health among young people in school settings. Young people enjoyed taking part in the research study and appreciated the autonomy provided in setting goals to reduce SSB intake. However, teachers found it difficult to attain the balance between didactic teaching and fostering collaboration and discussion in the classroom. Although we attempted to assess fidelity of the intervention, this was not feasible as the researchers were not permitted to observe in the classroom and the digital tape recorders were not used.

## Conclusion

This study highlighted that recruitment of study participants is a critical determinant of feasibility studies, which can directly limit the ability to assess practicality and acceptability of the intervention. Although schools can be a vehicle for promoting healthy behaviour change, the study has emphasised the significant barriers related to school, young people and parent recruitment and the challenges of teacher-student rapport. It signifies the importance of conducting feasibility studies prior to definitive trials.

## Supplementary material

10.1136/bmjph-2025-003961 online supplemental file 1

10.1136/bmjph-2025-003961 online supplemental file 2

10.1136/bmjph-2025-003961 online supplemental file 3

10.1136/bmjph-2025-003961 online supplemental file 4

10.1136/bmjph-2025-003961 online supplemental file 5

## Data Availability

Data are available upon reasonable request.
