# Supplementary material for: Feasibility and acceptability of a school-based Group Motivational Interviewing intervention to reduce sugar-sweetened beverages among young people in East London: DISS feasibility study
Source: BMJ Public Health. 2026 Apr 13;4(2):e003961. doi: 10.1136/bmjph-2025-003961 (PMC13084870; doi:10.1136/bmjph-2025-003961)
Supplement: online supplemental file 1 [file bmjph-4-2-s001.pdf]

Participant ID	
Date	

Young Person's Baseline Questionnaire

Thank you for agreeing to help us with this survey.

This is a questionnaire about you and some of your habits. Your answers will be looked at by the researchers and no-one else. They will not be seen by your parents. Because the questions being asked are about different subjects, some of them may seem strange to you. Please take your time to answer the questions and answer as honestly as you can. This is not a test and there are no right or wrong answers.

This questionnaire is made up of several questions organised in 5 sections.

Section 1: About You

1. **What is your date of birth? *(Write in)***

d	d	m	m	y	y

2. **What is your sex? *(Tick one box)***

☐Male
☐Female
☐Prefer not to say

3. **How would you describe your ethnicity? *(Tick one box)***

**White**	☐ English/Welsh/Scottish/ Northern Irish/British<br>☐ Irish<br>☐ Gypsy or Irish Traveller<br>☐ Any other white background
**Mixed/Multiple Ethnic Groups**	☐ White and Black Caribbean<br>☐ White and Black African<br>☐ White and Asian<br>☐ Any other mixed / multiple ethnic background
**Asian/Asian British**	☐ Indian<br>☐ Pakistani<br>☐ Bangladeshi<br>☐ Chinese<br>☐ Any other Asian background
**Black/ African/ Caribbean/Black British**	☐ African<br>☐ Caribbean<br>☐ Any other
**Other ethnic group**	☐ Arab<br>☐ Any other ethnic group

Section 2: Physical Activity

These questions are about the physical activity you do. Physical activity is anything you do that increases your heart rate and may make you get out of breath some of the time. Please include brisk walking or biking, dancing, skateboarding, doing sports, school activities during PE or during school breaks, and going to the gym.

4. Over the past 7 days, on how many DAYS were you physically active for a total of at least an hour per day? *(Write in)*		
5. OUTSIDE SCHOOL HOURS: How many HOURS a week do you usually exercise in your free time so much that you get out of breath or sweaty? *(Write in)*		
6. How many HOURS a day do you usually watch TV (including DVDs) in your free time? *(Write in)*	**Weekdays:**	
	**Weekends:**	
7. About how many HOURS a day do you usually play on a games console (e.g. Playstation, Nintendo DS), spend on social network sites (e.g. Facebook), or use a computer in general in your free time? **Note:** Do not include time spent on 'active' video gaming such as the Wii or Kinect. *(Write in)*	**Weekdays:**	
	**Weekends:**	

Section 3: Eating Habits

8. How many times a week do you usually eat or drink? *(Tick one box for each line)*

	Never	Less than once a week	Once a week	2-4 days a weeks	5-6 days a weeks	Once a day, every day	Every day, more than once
Fruits	☐	☐	☐	☐	☐	☐	☐
Vegetables	☐	☐	☐	☐	☐	☐	☐
Sweets (candy or chocolate)	☐	☐	☐	☐	☐	☐	☐
Coke or other soft drinks that contain sugar	☐	☐	☐	☐	☐	☐	☐

9. How often do you usually have breakfast (more than a glass of milk or fruit juice)? *(Tick one box for weekdays and one box for weekend)*

Weekdays		Weekends	
I never have breakfast during the week	☐	I never have breakfast during the weekend	☐
One day	☐		
Two days	☐	I usually have breakfast on only one day of the weekend (Saturday OR Sunday)	☐
Three days	☐		
Four days	☐	I usually have breakfast on both weekend days (Saturday AND Sunday)	☐
Five days	☐		

10. What do you think are the most popular sugary drinks among 12–13-year-olds? *(Write in)*

Section 4: Self-confidence and Motivation

11. How READY you are to make a change to sorts of drinks you have, on a scale of 1 to 5? (1= I'm not ready / 5= I'm ready) *(Write in)*	
12. How CONFIDENT are you that you can change the sorts of drinks you have, on a scale of 1 to 5? (1= I'm not confident / 5= I'm very confident) *(Write in)*	
13. What changes have you made? *(Write in)*	
14. How CONFIDENT are you to maintain these changes in the future, on a scale of 1 to 5? (1= I'm not confident / 5= I'm very confident) *(Write in)*	

Section 5: PSHE secondary education

We would like to know how you feel about your PSHE education (including statutory RSE and Health education).

**Your views on PSHE education (Please tick one box per line)**	**Strongly disagree**	**Disagree**	**Neutral**	**Agree**	**Strongly Agree**
15. I enjoy PSHE education lessons	☐	☐	☐	☐	☐
16. I learn a lot in PSHE education	☐	☐	☐	☐	☐
17. What we do in PSHE education is aimed too young for people of my age	☐	☐	☐	☐	☐
18. What we do in PSHE education is beyond what people of my age need	☐	☐	☐	☐	☐
19. I feel the scenarios and case studies we look at are relevant to people of my age	☐	☐	☐	☐	☐
20. I feel comfortable giving my views and opinions in PSHE education lessons	☐	☐	☐	☐	☐
21. Other students listen to my views and opinions in PSHE education lessons	☐	☐	☐	☐	☐
22. A wide range of different activities is used in PSHE education lessons	☐	☐	☐	☐	☐
23. I know how well I am doing in PSHE education and what I need to do to improve	☐	☐	☐	☐	☐
24. Enough time is given to PSHE education lessons	☐	☐	☐	☐	☐
25. Do you have any suggestion to improve your PSHE lessons? *(Please write in)*					

Thank You!
