# Supplementary material for: Feasibility and acceptability of a school-based Group Motivational Interviewing intervention to reduce sugar-sweetened beverages among young people in East London: DISS feasibility study
Source: BMJ Public Health. 2026 Apr 13;4(2):e003961. doi: 10.1136/bmjph-2025-003961 (PMC13084870; doi:10.1136/bmjph-2025-003961)
Supplement: online supplemental file 2 [file bmjph-4-2-s002.pdf]

Participant ID	
Date	

Young Person's Follow-Up Questionnaire (Intervention)

This questionnaire is made up of several questions organised in 5 sections.

Section 1: Physical Activity

These questions are about the physical activity you do. Physical activity is anything you do that increases your heart rate and may make you get out of breath some of the time. Please include brisk walking or biking, dancing, skateboarding, doing sports, school activities during PE or during school breaks, and going to the gym.

1. Over the past 7 days, on how many DAYS were you physically active for at least 1 hour? *(Write in)*		
2. OUTSIDE SCHOOL HOURS: How many HOURS a WEEK do you usually exercise in your free time so much that you get out of breath or sweaty? *(Write in)*		
3. How many HOURS a DAY do you usually watch TV (including DVDs) in your free time? *(Write in)*	**Weekdays:**	
	**Weekends:**	
4. About how many HOURS a DAY do you usually play on a games console (e.g. Playstation, Nintendo DS), spend on social network sites (e.g. Facebook), or use a computer in general in your free time? **Note:** Do not include time spent on 'active' video gaming such as the Wii or Kinect. *(Write in)*	**Weekdays:**	
	**Weekends:**	

Section 2: Eating Habits

5. How many times a week do you usually eat or drink? *(Tick one box for each line)*

	Never	Less than once a week	Once a week	2-4 days a weeks	5-6 days a weeks	Once a day, every day	Every day, more than once
Fruits	☐	☐	☐	☐	☐	☐	☐
Vegetables	☐	☐	☐	☐	☐	☐	☐
Sweets (candy or chocolate)	☐	☐	☐	☐	☐	☐	☐
Coke or other soft drinks that contain sugar	☐	☐	☐	☐	☐	☐	☐

6. **How often do you usually have breakfast (more than a glass of milk or fruit juice)?** ***(Tick one box for weekdays and one box for weekend)***

**Weekdays**		**Weekends**	
I never have breakfast during the week	☐	I never have breakfast during the weekend	☐
One day	☐		
Two days	☐	I usually have breakfast on only one day of the weekend (Saturday OR Sunday)	☐
Three days	☐		
Four days	☐	I usually have breakfast on both weekend days (Saturday AND Sunday)	☐
Five days	☐		

7. **What do you think are the most popular sugary drinks among 12–13-year-olds?** ***(Write in)***

Section 3: Self-confidence and Motivation

8. How READY you are to make a change to the sort of drinks you have on a scale of 1 to 5? (1= I'm not ready / 5= I'm ready) *(Write in)*	
9. How CONFIDENT are you that you can change the sorts of drinks you have, on a scale of 1 to 5? (1= I'm not confident / 5= I'm very confident) *(Write in)*	
10. What changes have you made? *(Write in)*	
11. How CONFIDENT are you to <u>maintain</u> these changes in the future, on a scale of 1 to 5? (1= I'm not confident / 5= I'm very confident) *(Write in)*	

Section 4: Support

12. How much do you feel that the PSHE sessions (on sugary drinks) helped you to change the way you eat, on a scale of 1 to 5? (1= unhelpful / 5= very helpful) *(Write in)*			
13. How much do you feel that the PSHE sessions (on sugary drinks) <u>helped</u> you to change your intake of sugary drinks, on a scale of 1 to 5? (1= unhelpful / 5= very helpful) *(Write in)*			
14. How much do you feel that the **DISSDash app** helped you to change your intake of sugary drinks, on a scale of 1 to 5? (1= unhelpful / 5= very helpful) *(Write in)*			
15. In the past 3 months, how much have your family and friends encouraged you to eat or drink healthily, on a scale of 1 to 5? (1= unhelpful / 5= very helpful) *(Write in)*	Family:		
	Friends:		
16. In the past 6 months, have you attended any weight management or healthy eating programmes or appointments? *(Write in)*		Y	N
**17.** If yes, what type or programme or appointment did you attend? (prompt for name)			

18. Do you have any further comments for the research team?

Section 5: PSHE secondary education

We would like to know how you feel about your PSHE education (including statutory RSE and Health education).

Your views on PSHE education (Please tick one box per line)	Strongly disagree	Disagree	Neutral	Agree	Strongly Agree
19. I enjoy PSHE education lessons	☐	☐	☐	☐	☐
20. I learn a lot in PSHE education	☐	☐	☐	☐	☐
21. What we do in PSHE education is aimed too young for people of my age	☐	☐	☐	☐	☐
22. What we do in PSHE education is beyond what people of my age need	☐	☐	☐	☐	☐
23. I feel the scenarios and case studies we look at are relevant to people of my age	☐	☐	☐	☐	☐
24. I feel comfortable giving my views and opinions in PSHE education lessons	☐	☐	☐	☐	☐
25. Other students listen to my views and opinions in PSHE education lessons	☐	☐	☐	☐	☐
26. A wide range of different activities is used in PSHE education lessons	☐	☐	☐	☐	☐
27. I know how well I am doing in PSHE education and what I need to do to improve	☐	☐	☐	☐	☐
28. Enough time is given to PSHE education lessons	☐	☐	☐	☐	☐
29. Do you have any suggestion to improve your PSHE lessons? *(Please write in)*					

Section 6: Acceptability and satisfaction

30. How satisfied were you with the number of sessions (on sugary drinks) you had? (Tick one box)

I would have preferred more sessions	☐
The number was exactly right	☐
I would have preferred fewer sessions	☐

31. How satisfied were you with the length of the sessions (on sugary drinks)? *(Tick one box)*

I would have preferred longer sessions	☐
The length was exactly right	☐
I would have preferred shorter sessions	☐

32. Read the following statements, which use rating scales with 5 places (Tick one box in each row)

**(Please tick one box per line)**	**Strongly disagree**	**Disagree**	**Neutral**	**Agree**	**Strongly Agree**
33. I felt I could talk openly with my PSHE teacher during the sessions (on sugary drinks)	☐	☐	☐	☐	☐
34. I felt that my PSHE teacher listened to me	☐	☐	☐	☐	☐
35. Overall, I felt the sessions (on sugary drinks) were useful to me	☐	☐	☐	☐	☐
36. I felt comfortable that the sessions (on sugary drinks) took place in the school	☐	☐	☐	☐	☐

37. What did you like about the sessions (on sugary drinks)? *(Write in)*

38. What would you change about the sessions (on sugary drinks)? *(Write in)*

39. Would you recommend the sessions (on sugary drinks) to a friend? *(Tick one box)*

Yes		No	

If **No**, please specify below why *(Write in)*

Thank You!
