# Supplementary material for: Feasibility and acceptability of a school-based Group Motivational Interviewing intervention to reduce sugar-sweetened beverages among young people in East London: DISS feasibility study
Source: BMJ Public Health. 2026 Apr 13;4(2):e003961. doi: 10.1136/bmjph-2025-003961 (PMC13084870; doi:10.1136/bmjph-2025-003961)
Supplement: online supplemental file 3 [file bmjph-4-2-s003.pdf]

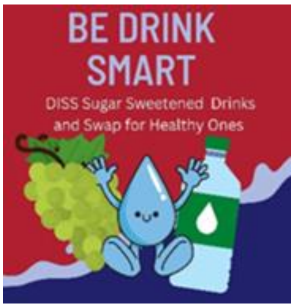

Queen Mary University of London
Medicine and Dentistry

Participant ID	
Date	

Parents/ Carers/ Guardians' Baseline Questionnaire

Dear parent/carer/guardian,

Thank you for agreeing in taking part in DISS (DISS Sugar Sweetened Drinks) to promote a reduction in sugary drinks consumption among young people and promote healthy living. We would be grateful if you could complete this questionnaire that will give us some background information about you and your family. The information you provide will be kept confidential. Your answers will be looked at by the researchers and no-one else. Please take your time to answer the questions and answer as honestly as you can. This is not a test and there are no right or wrong answers.

Thank you again for your help with this survey. Your views are very important to us.

This document is made up of several questions organised in 4 sections.

Section 1: About You

1. **What is your date of birth? *(Write in)***

d	d	m	m	y	y

2. **What is your sex? *(Tick one box)***

☐ Male
☐ Female
☐ Prefer not to say

3. **Which of these options best describes your employment status? *(Tick one box)***

Employed full-time	☐	Full-time student	☐
Employed part-time	☐	Full time	☐
Self-employed or freelance	☐	Long-term sick or disabled	☐
Unemployed / looking for work	☐	Carer	☐
Other (please describe)	☐		

---

4. **Which of these options best describes your employment status? *(Tick one box)***

No benefits	☐	Council Tax Benefit/ Reduction	☐
State Pension	☐	Income Support	☐
Job Seeker's Allowance	☐	Housing Benefit	☐
Universal Credit	☐	Disability Living Allowance	☐
Personal Independence Payment	☐	Employment Support Allowance	☐
Pension Credit	☐	Attendance Allowance	☐
Working Tax Credit	☐	Carer's Allowance	☐
Child Tax Credit	☐	Other state benefits (please describe)	☐

5. What is your highest qualification? *(Tick one box)*

No qualifications	☐	Higher National Diploma	☐
BTEC/ NVQ Level 1 or 2	☐	BTEC/ NVQ Level 4, 5 or 6	☐
University Degree	☐	GCSE or O Level	☐
Postgraduate	☐	A Level or AS Level	☐
BTEC/ NVQ Level 3	☐	Other qualification (please describe)	☐

6. Is your home…? *(Tick one box)*

Owned outright	☐	Rented	☐
Mortgaged	☐	Rent free (in a friend's or relative's property)	☐
Shared ownership (part rent and part mortgage)	☐		

Section 2: Eating Habits

7. At the moment, how often do you eat biscuits, pastries and cakes? *(Tick one box)*

At least once a day	☐	Once fortnight	☐
5-6 times a week	☐	Once a month	☐
3-4 times a week	☐	Less than once a month	☐
Once or twice a week	☐	Never	☐

8. At the moment, how often do you eat chocolates and sweets? *(Tick one box)*

At least once a day	☐	Once fortnight	☐
5-6 times a week	☐	Once a month	☐
3-4 times a week	☐	Less than once a month	☐
Once or twice a week	☐	Never	☐

9. At the moment, how often, on average do you have fizzy drinks, fruit juice, or soft drinks like squash, excluding diet or sugar-free drinks? *(Tick one box)*

At least once a day	☐	Once fortnight	☐
5-6 times a week	☐	Once a month	☐
3-4 times a week	☐	Less than once a month	☐
Once or twice a week	☐	Never	☐

10. At the moment, how often do you eat savoury snacks (crisps)? *(Tick one box)*

At least once a day	☐	Once fortnight	☐
5-6 times a week	☐	Once a month	☐
3-4 times a week	☐	Less than once a month	☐
Once or twice a week	☐	Never	☐

11. At the moment, how often do you eat Fruit and vegetables? *(Tick one box)*

At least once a day	☐	Once fortnight	☐
5-6 times a week	☐	Once a month	☐
3-4 times a week	☐	Less than once a month	☐
Once or twice a week	☐	Never	☐

12. Do you usually have sugar in hot drinks like tea and coffee? *(Tick one box)*

Yes ☐
No ☐
I don't drink tea or coffee ☐

Please tick one box per line:	Definitely disagree	Tend to disagree	Neutral	Tend to Agree	Definitely Agree
13. I get confused over what's supposed to be healthy and what isn't	☐	☐	☐	☐	☐
14. Small dietary changes, such as eating less fat or cutting down on sugar, can lead to benefits for my future health	☐	☐	☐	☐	☐
15. As long as you take enough exercise you can eat whatever you want	☐	☐	☐	☐	☐
16. The main reason for people to eat a more healthy diet is to lose weight	☐	☐	☐	☐	☐
17. What you eat makes a big difference to how healthy you are	☐	☐	☐	☐	☐

18. Some people may find it difficult to eat more healthily. Can you tell me please, what do you think would be the difficulties, if any, for you in trying to eat more healthily? *(Tick one box)*

No difficulties / already eat healthily ☐
Money / cost of healthy food ☐
Time constraints ☐
Time to prepare / cook food ☐
Healthy foods are too expensive ☐
Work commitments / hours ☐
Already eat healthily ☐
Giving up/cutting out sugar ☐
Don't like healthy food ☐
Giving up/cutting out chocolate ☐
Other (Please specify:) ☐

Section 3: Physical Activity

19. Which form of transport have you used most often in the last 4 weeks ending yesterday, apart from your journey to and from work? *(Tick one box)*

Car / motor vehicle ☐
Walk ☐
Public Transport ☐
Cycle ☐

20. In the last 4 weeks ending yesterday, how many hours did you watch TV, DVD or video per day? *(Tick one box in each line)*

	None	Less than 1 hour a day	1 to 2 hours a day	2 to 3 hours a day	3 to 4 hours a day	More than 4 hours a day
On a weekday before 6 pm	☐	☐	☐	☐	☐	☐
On a weekday after 6 pm	☐	☐	☐	☐	☐	☐
On a weekend day before 6 pm	☐	☐	☐	☐	☐	☐
On a weekend day after 6 pm	☐	☐	☐	☐	☐	☐

Section 4: Please provide the following information *(Write in)*

Mobile phone number	
Home phone number	
Email address	

This is the end of the questionnaire.

Thank you for your time!
