# Supplementary material for: Feasibility and acceptability of a school-based Group Motivational Interviewing intervention to reduce sugar-sweetened beverages among young people in East London: DISS feasibility study
Source: BMJ Public Health. 2026 Apr 13;4(2):e003961. doi: 10.1136/bmjph-2025-003961 (PMC13084870; doi:10.1136/bmjph-2025-003961)
Supplement: online supplemental file 4 [file bmjph-4-2-s004.pdf]

Queen Mary
University of London
Medicine and Dentistry

Please write in:

Participant ID	
Date	

Parents/ Carers/ Guardians'
Follow-Up Questionnaire (Intervention)

Thank you for agreeing to help us with this survey.

Please complete this short questionnaire. Your views are very important to us.

Thank you for your interest in taking part in DISS (**D**iss **S**ugar **S**weetened Drinks) to promote a reduction in sugary drinks consumption among young people and promote healthy living. We would be grateful if you could complete this questionnaire so we can check a few details just to make sure your family will be able to take part. It gives us some background information about you and your family. The information you provide will be kept confidential because we will remove the page with your name and the address on it. No member of your family will be identifiable to anyone outside of the research team. Your answers will be looked at by the researchers and no-one else. Please take your time to answer the questions and answer as honestly as you can. This is not a test and there are no right or wrong answers.

All responses to this survey are completely confidential.

Thank you for your time.

This document is made up of several questions organised in 3 questionnaires:

Questionnaire 1: Eating Habits

1. At the moment, how often do you eat biscuits, pastries and cakes? *(Tick one box)*

At least once a day		Once fortnight	
5-6 times a week		Once a month	
3-4 times a week		Less than once a month	
Once or twice a week		Never	

2. At the moment, how often do you eat chocolates and sweets? *(Tick one box)*

At least once a day		Once fortnight	
5-6 times a week		Once a month	
3-4 times a week		Less than once a month	

Once or twice a week		Never	

3. At the moment, how often, on average do you have fizzy drinks, fruit juice, or soft drinks like squash, excluding diet or sugar-free drinks? *(Tick one box)*

At least once a day		Once fortnight	
5-6 times a week		Once a month	
3-4 times a week		Less than once a month	
Once or twice a week		Never	

4. At the moment, how often do you eat savoury snacks (crisps)? *(Tick one box)*

At least once a day		Once fortnight	
5-6 times a week		Once a month	
3-4 times a week		Less than once a month	
Once or twice a week		Never	

5. At the moment, how often do you eat Fruit and vegetables? *(Tick one box)*

At least once a day		Once fortnight	
5-6 times a week		Once a month	
3-4 times a week		Less than once a month	
Once or twice a week		Never	

6. Do you usually have sugar in hot drinks like tea and coffee? *(Tick one box)*

Yes	
No	
I don't drink tea or coffee	

	Definitely disagree	Tend to disagree	Neutral	Tend to Agree	Definitely Agree
7. I get confused over what's supposed to be healthy and what isn't					
8. Small dietary changes, such as eating less fat or cutting down on sugar, can lead to benefits for my future health					
9. As long as you take enough exercise you can eat whatever you want					
10. The main reason for people to eat a more healthy diet is to lose weight					
11. What you eat makes a big difference to how healthy you are					

12. Some people may find it difficult to eat more healthily. Can you tell me please, what do you think would be the difficulties, if any, for you in trying to eat more healthily? *(Tick one box)*

No difficulties / already eat healthily	
Money / cost of healthy food	
Time constraints	
Time to prepare / cook food	
Healthy foods are too expensive	
Work commitments / hours	
Already eat healthily	
Giving up/cutting out sugar	
Don't like healthy food	
Giving up/cutting out chocolate	
Other (SPECIFY)	

Questionnaire 3: Physical Activity

1. Which form of transport have you used most often in the last 4 weeks ending yesterday, apart from your journey to and from work? *(Tick one box)*

Car / motor vehicle	
Walk	
Public Transport	
Cycle	

2. In the last 4 weeks ending yesterday, how many hours did you watch TV, DVD or video per day? *(Tick one box in each line)*

	None	Less than 1 hour a day	1 to 2 hours a day	2 to 3 hours a day	3 to 4 hours a day	More than 4 hours a day
On a weekday before 6 pm						
On a weekday after 6 pm						
On a weekend day before 6 pm						
On a weekend day after 6 pm						

Questionnaire 3: Acceptability and satisfaction

1. How satisfied were you with the number of sessions your child had? *(Tick one box)*

I would have preferred more sessions	
The number was exactly right	
I would have preferred fewer sessions	

2. How satisfied were you with the length of the sessions? *(Tick one box)*

I would have preferred longer sessions	
The length was exactly right	
I would have preferred shorter sessions	

4. What did you like about the sessions? *(Write in)*

5. What would you change about the sessions? *(Write in)*

6. Would you recommend the sessions to a friend? *(Tick one box)*

Yes		No	

7. If No, please specify below why *(Write in)*

This is the end of the questionnaire. Thank you for completing it.
