# Supplementary material for: Feasibility and acceptability of a school-based Group Motivational Interviewing intervention to reduce sugar-sweetened beverages among young people in East London: DISS feasibility study
Source: BMJ Public Health. 2026 Apr 13;4(2):e003961. doi: 10.1136/bmjph-2025-003961 (PMC13084870; doi:10.1136/bmjph-2025-003961)
Supplement: online supplemental file 5 [file bmjph-4-2-s005.pdf]

Queen Mary
University of London
Medicine and Dentistry

DISS TOPIC GUIDE

YOUNG PEOPLE

1. Introduction
- Thank you for taking part in the DISS Study. Your feedback is very valuable to us.
- Explain what we expect/need from them (including number of sessions)
- Reinforce the importance of confidentiality and anonymity
- Reassure that consent is an on-going process and they can withdraw at any time
- We would like you to open and tell us what you think
- We will report back to you at the end of the study
- Brief overview of the study (Problem, evidence, intervention, outcomes, stages)

2. Recruitment process

a. What do you think of the recruitment processes?

b. How did you find the recruitment process poster?

c. Prompts:

    1. What worked well?
    2. What were the challenges and what were the facilitators?
    3. How should we communicate our study to young people to increase their interest in participating in research studies in the future?
    4. Use of social media? Such as Facebook pages or class WhatsApp groups.
    5. What did you think of the incentives used (vouchers)?
    6. Any suggestions for improvements?

3. Communication

    a. How did the research team communicate with you?

    b. Prompts:

        i. Did you feel there was too much or too little just about right?

        ii. Were there any problems with communication? If yes, what were these and how could they be overcome?

        iii. Were there any problems with communication? If yes, what were these and how could they be overcome? Explore

4. Resources and PSHE Lessons

Prompts:

a. Impacts on young peoples' knowledge and behaviours

b. What went well within PSHE lessons?

c. What could we have done differently?

d. What didn't go so well?

e. What did you think of the mobile app? Any barriers or facilitators?

f. Acceptability of the intervention/resources

g. What would have helped?

5. Data collection and evaluation
   a. Experience of completing the survey questionnaires at baseline and follow-up
   b. Any facilitators and any barriers
   c. Did you think it was appropriate to measure young people in a school? Do you envisage any barriers? Were there any problems?
   d. What did you think of the measurement sessions?
   e. Any suggestions

6. Dissemination process
   a. How should we disseminate the findings from the study?
   b. Who should be involved?

7. Perception about research
   a. In your opinion how could we get your friends and family involved in similar research?
   b. Did you think the study was appropriate in a secondary school setting? Why?
   c. Did you enjoy being part of a research project?
   d. Would you be interested in taking part in research in the future? Why?
   e. Any suggestions
